# The Conceivable Role of Metabolic Syndrome in the Pathogenesis of Alzheimer’s Disease: Cellular and Subcellular Alterations in Underpinning a Tale of Two

**DOI:** 10.1007/s12017-025-08832-6

**Published:** 2025-05-16

**Authors:** Ekremah A. Alzarea, Hayder M. Al-Kuraishy, Ali I. Al-Gareeb, Athanasios Alexiou, Marios Papadakis, Olivia N. Beshay, Gaber El-Saber Batiha

**Affiliations:** 1 Hematopathology, Department of Pathology, College of Medicine, Jouf University, Sakaka, Saudi Arabia; 2 Department of Clinical Pharmacology and Medicine, College of Medicine, Mustansiriyah University, Baghdad, Iraq; 3 Jabir Ibn Hayyan Medical University, Al-Ameer Qu./Najaf-Iraq, PO.Box13, Kufa, Iraq; 4 University Centre for Research & Development, Chandigarh University, Chandigarh-Ludhiana Highway, Mohali, Punjab Australia; 5 Department of Research & Development, Funogen, Athens, Greece; 6 Department of Surgery II, University Hospital Witten-Herdecke, University of Witten-Herdecke, Heusnerstrasse 40, 42283 Wuppertal, Germany; 7 Department of Biochemistry, Faculty of Pharmacy, Minia University, Minia, 61519 Egypt; 8 Department of Pharmacology and Therapeutics, Faculty of Veterinary Medicine, Damanhour University, Damanhour, 22511 AlBeheira Egypt

**Keywords:** Metabolic syndrome, Neuroinflammation, Alzheimer’s disease, Insulin resistance

## Abstract

Alzheimer’s disease (AD)is an age-related neurodegenerative disease characterized by memory decline and cognitive impairment .AD is common in people aged > 65 years, though most of AD cases are sporadic, which accounts for 95%, and 1–5% of AD is caused by familial causes . The causes of AD are aging, environmental toxins, and cardiometabolic factors that induce the degeneration of cholinergic neurons. It has been shown that the metabolic syndrome which is a clustering of dissimilar constituents including insulin resistance (IR), glucose intolerance, visceral obesity, hypertension, and dyslipidemia is implicated in the pathogenesis of AD. Metabolic syndrome disapprovingly affects cognitive function and the development in AD by inducing the development of oxidative stress, neuroinflammation, and brain IR. These changes, together with brain IR, impair cerebrovascular reactivity causing cognitive impairment and dementia. Nevertheless, the fundamental mechanism by which metabolic syndrome persuades AD risk is not entirely explicated. Accordingly, this review aims to discuss the connotation between metabolic syndrome and AD. In conclusion, metabolic syndrome is regarded as a possible risk factor for the initiation of AD neuropathology by diverse signaling pathways such as brain IR, activation of inflammatory signaling pathways, neuroinflammation, defective proteostasis, and dysregulation of lipid mediators.

## Introduction

Metabolic syndrome is a complex disorder defined by a cluster of interconnected factors such as glucose intolerance , insulin resistance (IR) , obesity and hypertension that increase the risk of cardiovascular diseases and type 2 diabetes mellitus (T2D) (Fahed et al., 2022; Lann & LeRoith, 2007; Grundy, 2016). According to the International Diabetes Federation and the American Heart Association, the presence of 3 components of metabolic syndrome is considered as diagnostic criteria for the metabolic syndrome (Weihe & Weihrauch-Blüher, 2019). The causes of the metabolic syndrome are sedentary life, high carbohydrate/fat diet, alcoholism, sleep disorders, aging, and genetic factors (Dimitrijevic et al., 2019; Nilsson et al., 2019; H. H. Wang et al., 2020). For example, chronic stress can promote the development of metabolic syndrome through dysregulation of the hypothalamic-pituitary axis, leading to an increase in circulating cortisol levels, which cause IR, T2D, and visceral obesity (Pasquali et al., 2006). Despite epidemiological data revealing extensive correlations between chronic stress exposure and metabolic disease, the underlying mechanisms responsible are still unknown. Mechanistic investigations of the effects of chronic social stress are being performed in rodent and nonhuman primate models, and the phenotypic findings are similar with those in humans. The benefit of these models is that probable neurological pathways may be investigated and interventions to treat or prevent disorders may be developed and evaluated. Additionally, circadian disturbance and metabolic disorders like T2D may enhance vulnerability to additional stressors or act as stressors themselves (Tamashiro et al., 2011). Chronic stress increases the nocturnal level of cortisol in obesity, as evidenced by animal model studies (Pasquali et al., 2006) suggesting that dysregulation of the hypothalamic-pituitary axis is implicated in the development of metabolic syndrome. Furthermore, aging enhances obesity and the progression of metabolic syndrome due to the development of skeletal muscle IR (Shou et al., 2020). In addition, IR can induce the development of metabolic syndrome through the induction of glucolipotoxicity and triggering of pro-inflammatory response (Gallagher et al., 2010). Metabolic syndrome provokes central and peripheral IR by increasing the production of hepatic very low-density lipoprotein, pro-inflammatory cytokines, and inhibition of the release of endothelial nitric oxide (H. Al-kuraishy et al., 2021; H. M. Al-kuraishy et al., 2023; Al-Naimi et al., 2019; Gallagher et al., 2010; Yanai et al., 2021). Despite these findings, there remains a question about whether IR is the cause or the outcome of metabolic syndrome. In pathophysiological terms, IR and compensatory hyperinsulinemia are causally related to each of glucose intolerance, dyslipidaemia, high blood pressure, and vascular dysfunction. Nevertheless, IR/hyperinsulinemia alone cannot produce these abnormalities; thus, additional pathogenic factors like β-cell dysfunction for glucose intolerance are needed. Whereas apparent diabetes, clinical hypertension, and frank dyslipidaemia are frequently present in the same patient, a subclinical condition with a different, likely etiology and established power as a risk factor is yet to be recognized (Ferrannini, 2006).

**Fig. 1:**
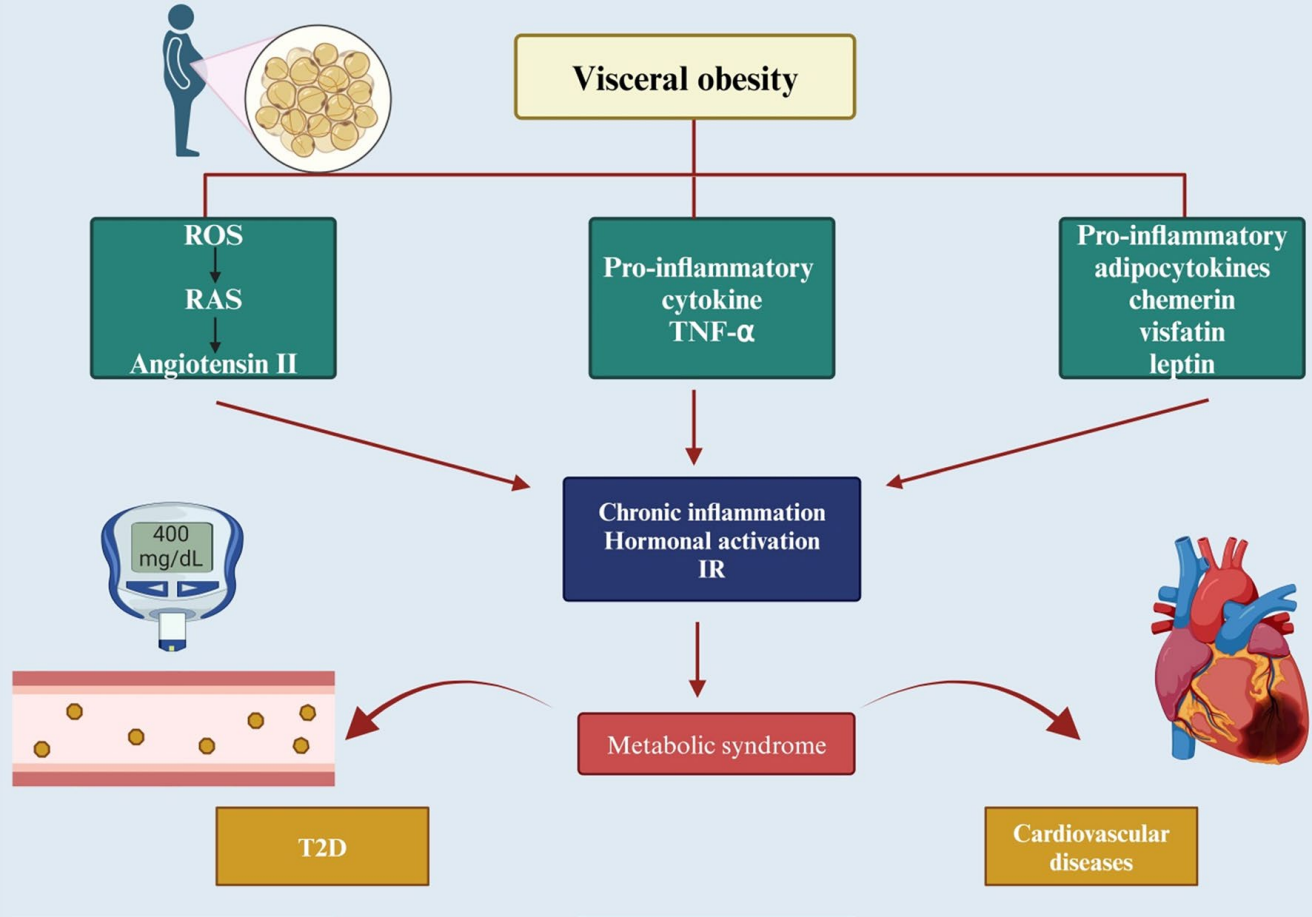
The link between visceral obesity and metabolic syndrome: Visceral obesity, by inducing the release of pro-inflammatory adipocytokines (chemerin, visfatin, and leptin), pro-inflammatory cytokine (TNF-α), and activating the production of reactive oxygen species (ROS), promotes the activation of RAS, leading to the development of chronic inflammation, hormonal activation, and IR. These changes promote the development of metabolic syndrome, which is interrelated with the development of T2D and cardiovascular diseases. “Created in BioRender. Alexiou, A. (2025) https://BioRender.com/o57y182”

Furthermore, visceral obesity which is the most prevalent may be the main pathology o the metabolic syndrome. Although abdominal obesity or visceral obesity is considered to be one of the components of metabolic syndrome and to have an important role in a cluster of cardiovascular risks, there is no consensus about the definition and diagnostic criteria for this syndrome, probably because there is considerable disagreement about the location and definition of abdominal obesity or visceral obesity. The importance of diagnosing metabolic syndrome, in which visceral fat accumulation plays an essential role in the development of multiple risk factors, should be emphasized because lifestyle modification for the reduction of visceral fat may be very effective for the reduction of risks of this type, namely metabolic syndrome in the narrow sense (Alkuraishy & AlGareeb, 2016; Koyama et al., 2020). Body fat distribution, particularly visceral adipose tissue accumulation, is a primary correlate of a set of diabetogenic, atherogenic, prothrombotic, and pro-inflammatory metabolic abnormalities associated with malfunctioning adipocytes and dysregulated adipocytokines production. Visceral fat reduction by health promotion strategies that use risk factor-oriented interventions may be successful in lowering atherosclerotic cardiovascular disorder events in people with metabolic syndrome (Shah et al., 2014). However, it is clear that the accumulation of visceral/ectopic fat is a major contributor to cardiovascular and metabolic risk above and beyond the BMI, implementation of fat distribution assessment into clinical practice remains a challenge. Anthropometric indices of obesity are easily implemented but newer imaging-based methods offer improved sensitivity and specificity for measuring specific depots. Lifestyle, pharmacologic, and surgical interventions allow a multidisciplinary approach to overweight/obesity that may improve outcomes and align with a public health message to combat the growing epidemic of obesity worldwide and build healthier lives, free of cardiovascular diseases (Koyama et al., 2020). In particular, visceral obesity has higher hyper-lipolytic activity and prothrombotic state increasing the risk of cardiovascular complications such as endothelial dysfunction, atherosclerosis, hypertension, IR, and T2D (Silveira et al., 2021). Visceral obesity promotes the release of the pro-inflammatory cytokine tumor necrosis factor alpha (TNF-α) (Kern et al., 2018). Low-grade inflammatory status in visceral obesity can cause IR and the development of T2D (Hildebrandt et al., 2023). Pro-inflammatory molecules produced by adipose tissue have been implicated in the risk of cardiovascular disease in obesity. The expression of critical pro-inflammatory genes is substantially higher in subcutaneous adipose tissue than in visceral adipose tissue in individuals with morbid obesity. The variability in circulating levels of pro-inflammatory cytokines due to underlying gene expression in subcutaneous adipose tissue but not in visceral adipose tissue. These results point to that abdominal subcutaneous adipose tissue contributes more than visceral adipose tissue to the pro-inflammatory milieu associated with severe obesity. Furthermore, visceral obesity through induction of oxidative stress and inflammatory reaction promote the activation of renin-angiotensin system (RAS) which implicated in the development of IR and progression of T2D and hypertension (Alexandre-Santos et al., 2022; Ramalingam et al., 2017). Notably, visceral adipose tissuehas RAS that can produce angiotensin II, resulting in systemic inflammation and oxidative stress with subsequent cardiovascular complications.All components of the RAS are expressed in and have independent regulation of adipose tissue. This local adipose RAS exerts important auto/paracrine functions in modulating lipogenesis, lipolysis, adipogenesis as well as systemic and adipose tissue inflammation. (Alexandre-Santos et al., 2022; Strazzullo et al., 2003). In addition, visceral adipose tissue produces adipocytokines such as leptin which implicated in the metabolic syndrome. Similarly, visceral fat is an important site for IL-6 secretion and provide a potential mechanistic link between visceral fat and systemic inflammation in people with abdominal obesity (Kumari et al., 2019). Therefore, visceral adiposity plays a major role in the development of metabolic syndrome (Fig. 1).

Furthermore, metabolic syndrome affects the cognitive function and induces the development of neurodegenerative disorders s by inducing oxidative stress, neuroinflammation, and brain IR (Arshad et al., 2018). Metabolic syndrome is a risk factor for neurological disorders such as stroke, depression and AD. The molecular mechanism underlying the mirror relationship between metabolic syndrome and neurological disorders is not fully understood. However, it is becoming increasingly evident that all cellular and biochemical alterations observed in metabolic syndrome like impairment of endothelial cell function, abnormality in essential fatty acid metabolism and alterations in lipid mediators along with abnormal insulin/leptin signaling may represent a pathological bridge between metabolic syndrome and neurological disorders (Raffaitin et al., 2009; Vanhanen et al., 2006; Yates et al., 2012). Though, the fundamental mechanisms by which metabolic syndrome augment AD risk are not entirely clarified. Consequently, this review tries to discuss the link between metabolic syndrome and AD.

## Outline the Pathogenesis of AD

AD is the leading cause of dementia and is characterized by a progressive decline in cognitive function, which typically begins with deterioration in memory (H. M. Al-kuraishy et al., 2023). AD is common in people aged > 65 years (Reiss et al., 2024). However, the development of AD below the age of 65 years is called early-onset AD (Tellechea et al., 2018). Most of AD cases are sporadic, which accounts for 95%, and 1–5% of AD is caused by familial causes (Andrade-Guerrero et al., 2023). The causes of AD are aging, environmental toxins, and cardiometabolic factors that induce the degeneration of cholinergic neurons (Breijyeh & Karaman, 2020; Comaposada-Baró et al., 2023). The mechanisms underlying the neuropathological changes in AD remain unclear. Genetic factors are mainly implicated in the development of early-onset AD; however, environmental factors are mostly involved in the development of late-onset AD (Dai et al., 2018; Wainaina et al., 2014).

**Fig. 2:**
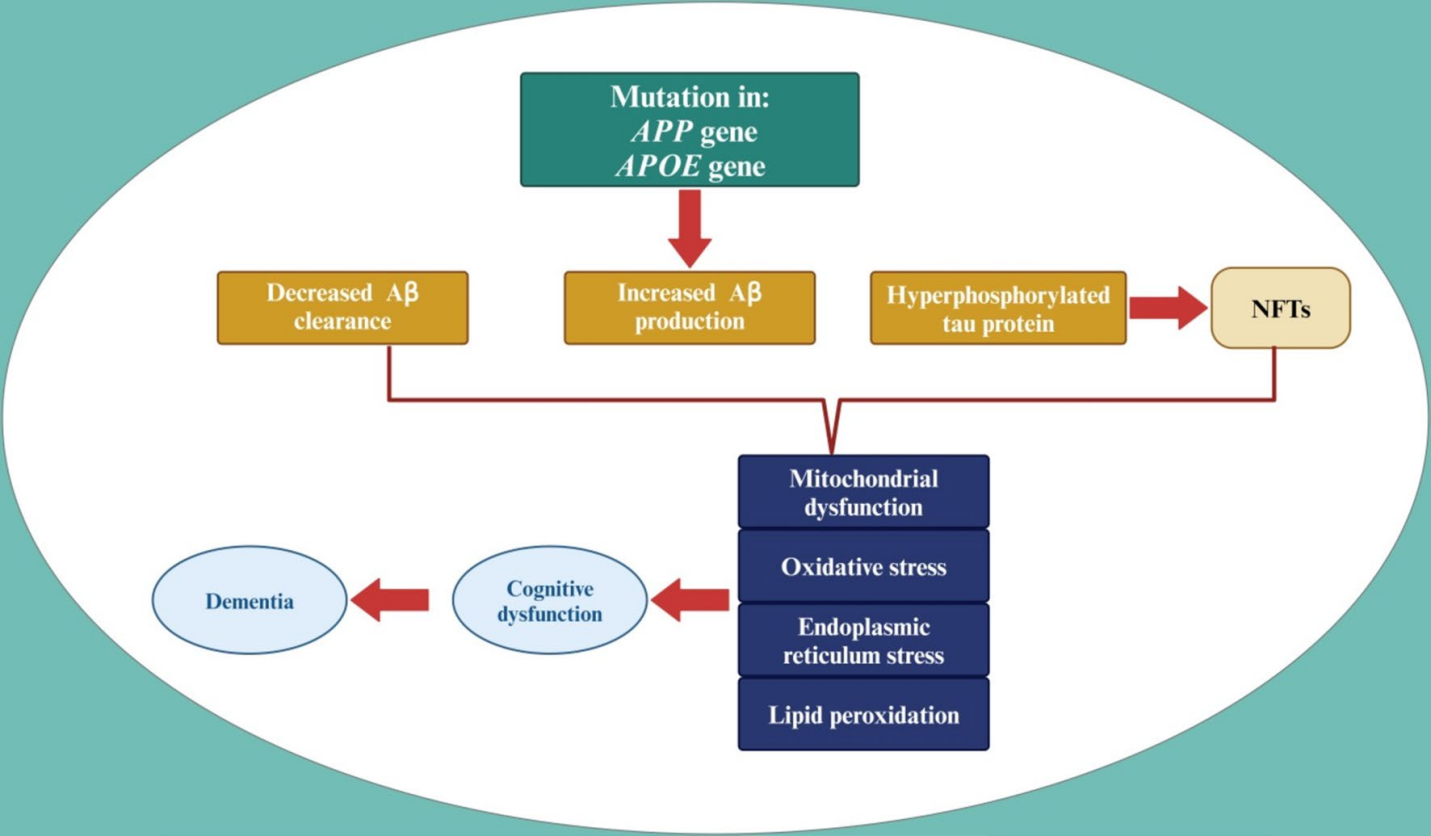
Pathogenesis of AD: Overproduction of Aβ due to mutations in APP and ApoE genes, and/or decreasing of Aβ clearance, and tau protein hyperphosphorylation and formation of NFTs, leads to mitochondrial dysfunction, oxidative stress, endoplasmic reticulum stress, and lipid peroxidation. These neuropathological changes provoke the development of cognitive dysfunction and the progression of dementia. “Created in BioRender. Alexiou, A. (2025) https://BioRender.com/p32l942”

There are several theories and so far for the development of AD, none of them is completely accepted (Ali et al., 2024; Ju & Tam, 2022). The neuropathological hallmarks of the AD brain are diffuse and neuritic extracellular amyloid plaques-which are frequently surrounded by dystrophic neurites-and intracellular neurofibrillary tangles. These hallmark pathologies are often accompanied by the presence of reactive microgliosis and the loss of neurons, white matter and synapses (Alsubaie et al., 2022; Yun Zhang et al., 2023). Normally, both Aβ and tau proteins are involved in the regulation of neurotransmitters release and the stability of neuronal microtubules and axonal transport, respectively. In addition, Aβ is eliminated through neuronal autophagy and blood–brain barrier (BBB) into the systemic circulation where it metabolized by the liver and excreted by the kidney. Aβ is generated from amyloid precursor protein (APP) by amyloidogenic pathway which is augmented by aging (Gali et al., 2019; Sehar et al., 2022). However, in healthy and young subjects, most of APP processing is through a non-amyloidogenic pathway which generates the neuroprotective soluble APP alpha (sAPPα). Therefore, overproduction of Aβ due to mutation in the *APP* gene or defective in the clearance of Aβ promotes AD neuropathology by inducing the accumulation of Aβ which induces hyperphosphorylation of tau protein (Al-Kuraishy et al., 2023; Galvão et al., 2019). Both insoluble Aβ and hyperphosphorylated tau proteins trigger series of reactions leading to mitochondrial dysfunction, oxidative stress, endoplasmic reticulum stress, and neural lipid peroxidation (Ajoolabady et al., 2022; AlAnazi et al., 2023; Lloret et al., 2015; Reddy & Oliver, 2019; Sharma & Kim, 2023). Remarkably, hyperphosphorylated tau protein is more intricate in AD neuropathology than Aβ (Muralidar et al., 2020). These neuropathological changes provoke synaptic dysfunction and neuronal apoptosis, resulting in the development of cognitive impairment and dementia (Plascencia-Villa & Perry, 2023). Furthermore ApoE4 is implicated in the pathologic process of AD.ApoE isoforms exert a central role in controlling the transport of brain lipid, neuronal signaling, mitochondrial function, glucose metabolism, and neuroinflammation. Regardless of widespread indispensable studies, the appropriate function of APOE in AD etiology stays ambiguous. Existing proof recommends that the disparate outcomes of ApoE isoforms on Aβ accretion and clearance have a distinct function in AD pathogenesis. ApoE-lipoproteins combine diverse cell-surface receptors to transport lipids and moreover to lipophilic Aβ peptide, that is believed to begin deadly events that generate neurodegeneration in the AD (Uddin et al., 2019). ApoE is a functional protein improves the integrity of neurons and synaptic plasticity by reducing oxidative stress. However, mutation of the *ApoE* gene and the production of aberrant ApoE can induce the overproduction of Aβ and enhance the formation of amyloid plaques, a hallmark of AD. The ApoEε4 allele is the most significant genetic risk factor for sporadic AD, while the ApoEε2 allele is the strongest genetic protective factor, as evidenced by numerous large-scale genome-wide association studies and meta-analyses (Emrani et al., 2020; Serrano-Pozo et al., 2021; Uddin et al., 2019). In addition, ApoE is also interacting with tau protein in the presence of Aβ, which results in neurodegeneration (Loch et al., 2023). ApoE has great influence in tau pathogenesis, tau-mediated neurodegeneration, and neuroinflammation, as well as α-synucleinopathy, lipid metabolism, and synaptic plasticity despite the presence of Aβ pathology. ApoE4 shows the deleterious effect for AD while the lack of ApoE4 is defensive. Therapeutic strategies primarily depend on APOE suggest to lessen the noxious effects of ApoE4 and reestablish the protective aptitudes of ApoE. This appraisal represents the critical interactions of APOE and AD pathology, existing facts on ApoE levels in the central nervous system (CNS), and the credible active stratagems for AD therapy by aiming ApoE. ApoE promotes neuronal injury mainly in familial AD (Butterfield & Mattson, 2020). Collectively, AD neuropathology is multifaceted (Fig. 2).

##  Search Strategy

We considered both PubMed and Google Scholar to identify studies relevant to this review. All publications included human subjects and had, at a minimum, to control for the effects of adiposity or metabolic dysfunction. For instance, studies examining the effects of adiposity on cognition had to either exclude patients with a diagnosis of T2D or had to control for the effects of blood glucose, insulin, or HOMA-IR. Publications examining the effects of metabolic dysfunction on cognition had to either exclude participants with obesity or control for one of the many anthropometric features of adiposity, including body weight, BMI, body fat %, waist/hip ratio, and waist circumference. The descriptors using the MeSH database are as follows (Alzheimer’s disease AND Metabolic syndrome), (Alzheimer’s disease AND Insulin resistance), (Alzheimer’s disease AND Obesity), (Alzheimer’s disease AND Hypertension), and (Alzheimer’s disease AND Dyslipidemia). All articles according to the inclusion criteria were estimated. However, reviews, letters, and articles other than English language were excluded.

## Metabolic Syndrome and Risk of AD

Growing evidence supports the concept that AD is fundamentally a metabolic disease with molecular and biochemical features that correspond with peripheral IR. Brain IR and its consequences can readily account for most of the structural and functional abnormalities in AD. However, disease pathogenesis is complicated by the fact that AD can occur as a separate disease process, or arise in association with systemic IR diseases including diabetes, obesity, and metabolic syndrome. Whether primary or secondary in origin, brain insulin/IGF resistance initiates a cascade of neurodegeneration that is propagated by metabolic dysfunction, increased oxidative and ER stress, neuro-inflammation, impaired cell survival, and dysregulated lipid metabolism (Kim & Feldman, 2015). Mounting evidence reveals that metabolic syndrome is associated with the development and progression of AD; nevertheless, the factors underlying this link have yet to be discovered. The central component of metabolic syndrome, IR, is the fundamental relationship between metabolic syndrome and AD. In the CNS, insulin plays significant roles in learning and memory, and AD patients have impaired insulin signaling that is identical to that noticed in metabolic syndrome (Kim & Feldman, 2015). Findings from a preclinical study showed that metabolic syndrome provokes the development of neuroinflammation in the white matter of the AD rat brains. This outcome reveals that white matter neuroinflammation could be one of the probable processes underlying the early interaction of metabolic syndrome with the development of mild cognitive impairment and pre-AD, as well as one of the early brain pathologies that contribute to cognitive deficits observed in mild cognitive impairment and AD (Ivanova et al., 2020). In addition, a high-fat diet can induce AD-like pathology in an animal model by disrupting hippocampal-dependent learning and memory processes, particularly those involving spatial memory. Consumption of a high-fat diet resulted in a decrease in mRNA expression of tight junction proteins, notably claudin proteins in the BBB, suggesting that hippocampal function may be specifically subject to disruption by a high-fat diet, and this disruption may be correlated to impaired integrity of the BBB (Kanoski et al., 2010). In addition, a high-sucrose diet produced fasting normoglycemia associated with hyperinsulinemia and hypertriglyceridemia in rats, resulting in poorer performance in hippocampal-dependent short- as well as long-term spatial memory. Prominently, high-sucrose animals showed elevated hippocampal levels of AMPA and NMDA receptors, while the levels of synaptophysin (protein markers linked to nerve terminals) and oxidative stress/inflammation remained unchanged. These data demonstrate that a prediabetic state evoked by a high-sucrose diet results in short- and long-term spatial memory deficits associated with changes in hippocampal glutamatergic neurotransmission (Soares et al., 2013). Hyperinsulinemia and IR were proposed more than 30 years ago to be important contributors to elevated blood pressure (BP) associated with obesity and the metabolic syndrome, also called syndrome X. Support for this concept initially came from clinical and population studies showing correlations among hyperinsulinemia, IR, and elevated BP in individuals with metabolic syndrome. Therefore, metabolic dysregulation caused by obesity and prediabetes provokes the development of cognitive impairment and AD. Likewise, T2D and metabolic syndrome accelerate AD incidence when they coexist in patients with mild cognitive impairment, whereas regular use of antidiabetic and antihyperlipidemic agents can reduce AD in patients with metabolic syndrome. AD risk factors identified by genome-wide association studies (GWAS) have strongly suggested the role of microglia in AD pathogenesis. Microglial dysregulation is caused not only by CNS-intrinsic factors but also by systemic changes. Metabolic syndrome appears to cause brain mitochondrial dysfunction through a defective NAD/SIRT1 pathway. Intense cardiovascular risk reduction and lifestyle changes for individuals with mild cognitive impairment and T2D, prediabetes, or metabolic syndrome could be beneficial to reduce the occurrence of dementia in this group at greatest risk (Pal et al., 2018). Moreover, hyperglycemia promotes elevated peripheral utilization of insulin, which reduces insulin transport into the brain. Molecular mechanisms have been demonstrated to protect CNS neurons from Aβ derived diffusible ligands (ADDLs), which cause synaptic deterioration and contribute to AD memory failure. The protective mechanism does not include simple competition between ADDLs and insulin, instead involving signaling dependent down-regulation of ADDL-binding sites. Dysfunctional insulin signaling subjects neurons to energy deficiency and susceptibility to oxidizing or other metabolic insults and defects synaptic plasticity. The interaction between Aβ and tau proteins might induce neuronal loss. Hyperinsulinemia and total insulin deficiency lead to increased tau phosphorylation, resulting in an imbalance of insulin-regulated tau kinases and phosphatases. On the other hand, amyloid peptide accumulation is a crucial step in the pathologic process of AD. Chronic hyperinsulinemia may trigger inflammatory responses and elevate biomarkers of oxidative stress. Moreover, insulin shows to act as neuromodulator, affecting release and reuptake of neurotransmitters, and enhancing learning and memory (Bosco et al., 2011).

Of interest, metabolic syndrome is correlated with an elevated risk of cerebrovascular disorders, involving cerebral ischemia. Microvascular dysfunction is a significant aspect underlying the pathogenesis of cerebrovascular illness. It has been demonstrated that animals with metabolic syndrome are more vulnerable to changes in the cerebral microcirculation by promoting endothelial dysfunction and oxidative stress (Obadia et al., 2017). The APP processing is implicated in the pathologic process of several neurodegenerative diseases. It has been shown that alterations in the expression of the primary participants in the processing of APP in neurons and astrocytes follow photothrombotic stroke (Sharifulina et al., 2022). Cai et al. demonstrated that chronic cerebral hypoperfusion contributes to cognitive impairment and alters the amyloidogenic and non-amyloidogenic pathways of APP processing through increasing the activity of β-secretase/γ-secretase and α-secretase, respectively. The non-amyloidogenic pathway is unable to mitigate the amyloidogenic pathway’s damaging impact in the process of chronic cerebral hypoperfusion, which increases amyloid-beta pathogenesis (Cai et al., 2017). These findings reveal a possible mechanistic link between AD and vascular factors.

Interestingly, exaggerated pro-inflammatory cytokines in metabolic syndrome can induce the formation of Aβ via the expression of *APP* gene in PSV2UTR-CAT-transfected cells (Lahiri et al., 2003). Emerging evidence suggests that aberrant signaling in a brain-periphery metabolic axis is linked to AD pathogenesis. The stimulation of pro-inflammatory pathways in the brain, particularly the interleukin-6 (IL-6) pathway, may be a prevalent connection between memory dysfunction and metabolic changes in AD. Postmortem AD brains showed elevated levels of IL-6 and suppressor of cytokine signaling 3. Furthermore, the IL-6 pathway was activated in the hypothalamus and hippocampus of AD mice. Neutralization of IL-6 and suppression of the signal transducer and activator of transcription 3 signaling in the brains of AD mouse models reduced memory impairment and peripheral glucose intolerance and normalized plasma levels of IL-6. Together, IL-6 and other pro-inflammatory cytokines are correlated to cognitive impairment and peripheral metabolic changes in AD. Consequently, focusing on pro-inflammatory IL-6 signaling may be an approach for reducing memory impairment and metabolic changes in the metabolic syndrome (Lyra E Silva et al., 2021).

In clinical setting, Hishikawa et al. illustrated that AD patients with metabolic syndrome had greater cognitive impairment compared to AD without metabolic syndrome. Besides, vascular endothelial dysfunction and brain IR are more intense in AD patients with metabolic syndrome before the appearance of brain white matter changes (Hishikawa et al., 2016). Thus, greater cognitive and affective decline occurs in patients with AD-metabolic syndrome than in those without. In addition, IR and vascular endothelial dysfunction are strongly correlated with AD-metabolic syndrome before pathological white matter changes can be observed. Interestingly, AD risk is more in women with metabolic syndrome than men, proposing that women are vulnerable to the harmful effects of metabolic syndrome (Vanhanen et al., 2006) due to hormonal changes and the risk of gestational diabetes (Ou et al., 2023; Pathirana et al., 2021). Conversely, Raffaitin et al. clarified that metabolic syndrome increases risk of vascular dementia but not AD risk (Raffaitin et al., 2009). The identified link between high triglycerides, T2D, and vascular dementia reinforces the importance of detecting and treating vascular risk factors in older people to reduce the likelihood of clinical dementia. Nevertheless, several limitations may affect the interpretation of these results. Selective survival might explain some paradoxical results in the oldest elderly subjects, in whom metabolic syndrome was found to be associated with slower cognitive decline. The association between metabolic syndrome and dementia might indeed be underestimated as a result of censoring for death since subjects with metabolic syndrome are more likely to die from cardiovascular disease before developing dementia. It would be interesting to explore the causes of death in patients with metabolic syndrome. A systematic review and meta-analysis disclosed a significant correlation between the components of metabolic syndrome and AD incidence (Zuin et al., 2021). These findings highlighted that metabolic syndrome is associated with high AD risk. Nevertheless, the exact component of metabolic syndrome that interconnected with AD was not completely interpreted.

On the other hand, there is increasing evidence that AD may be a widespread systemic disorder, suggesting that peripheral organs might be affected by pathological pathways occurring in this neurodegenerative illness. Consistently, AD in transgenic mice is associated with systemic metabolic disorders as evident by the presence of significant changes in different metabolites including sphingolipids, steroids, and acylcarnitines compared to the wild mice. Indeed, systemic oxidative stress, bioenergetics failure, impairment of gluconeogenesis, and metabolism of branched amino acids are present in AD models (González-Domínguez et al., 2015). This finding clearly supports the hypothesis that AD may be considered as a systemic disorder. In a clinical setting, a case–control study indicated that AD patients had a higher waist circumference, impaired glucose tolerance, and dyslipidemia, suggesting that AD may be a potential risk factor for the development of metabolic syndrome (Razay et al., 2007). A case–control study found that serum Aβ42 levels were positively correlated with the blood pressure, body mass index, and lipid profile in patients with metabolic syndrome (K. Li et al., 2024). However, plasma Aβ42 levels are reduced in AD patients in comparison with controls due to immune tolerance (W. Xu et al., 2008). Therefore, peripheral Aβ42 is regarded as potential biomarker of both AD and metabolic syndrome. Hence, AD may be a possible risk factor in the development and progression of metabolic syndrome.

Importantly, genetic factors may be a risk for both metabolic syndrome and AD (Abou Ziki & Mani, 2016; Tanzi, 2012). It is well known that *APOE* ε4 allele is a potential genetic risk factor in the development of AD (Emrani et al., 2020; Uddin et al., 2019). In addition, polymorphism of APOE ε4 which involved in lipid metabolism is associated with risk of metabolic syndrome (Povel et al., 2011). A population study illustrated that genetic variants at the *APOE* locus predict the development of metabolic syndrome (Yeh et al., 2022). Emerging evidence reveals that exposure to environmental toxicants in early life is linked to the development of metabolic syndrome later in life via epigenetic pathways (G. Wang et al., 2014a, 2014b). These verdicts highlighted that environmental and genetic factors are involved in the pathologic process of AD and metabolic syndrome.

It has been established that high-density lipoprotein (HDL) serum level is positively correlated with cognitive performance and memory function (Bates et al., 2017; Crichton et al., 2014). A cross-sectional study revealed that subjects with HDL levels higher than 60 mg/dl had better cognitive outcomes compared to those with low HDL levels (Crichton et al., 2014). A prospective cohort study illustrated that HDL serum level > 55 mg/dL is associated with lower AD risk (Reitz et al., 2010). Thus, low HDL serum level in metabolic syndrome is correlated with high AD risk (Michikawa, 2003). Increasing epidemiological and biological evidence indicates an association between blood cholesterol levels and the development of AD, as well as the prospective therapeutic value of statins for AD and mild cognitive impairment, while other lines of evidence reveal conflicting conclusions. Cholesterol interacts with Aβ in a reciprocal way, in which cellular cholesterol levels regulate Aβ synthesis, while Aβ changes cholesterol dynamics in neurons, resulting in tauopathy (Michikawa, 2003). Of note, HDL transports Aβ in cerebrospinal fluid (CSF) and plasma, which may eliminate excess peptides from the brain (Kontush & Chapman, 2008). Therefore, the reduction of HDL serum in metabolic syndrome might be a potential risk factor for the development and progression of AD. Moreover, hypertriglyceridemia which is an important component of metabolic syndrome is also implicated in AD neuropathology (Watts & Mamo, 2021). A population-based cohort study disclosed that moderate hypertriglyceridemia is connected with the development of dementia (Nordestgaard et al., 2021). Findings from experimental studies demonstrated that triglyceride-rich lipoproteins enhance the delivery of Aβ from the liver to the brain through uptake at the CSF level (James et al., 2003; Wellington & Frikke-Schmidt, 2016).

Furthermore, IR and hyperglycemia which are commonly associated with metabolic syndrome are also implicated in AD neuropathology (T. Li et al., 2021; Mikhail, 2009). IR may have a significant impact on the development of hyperglycemia as well as dyslipidemia, which can further aggravate IR. The contribution of IR in hypertension tends to be less strong than its effect in causing hyperglycemia and dyslipidemia. Additionally, obesity initiates or exacerbates IR. Similar to insulin resistance, obesity is not universal in the metabolic syndrome, and many obese individuals do not exhibit metabolic abnormalities (Mikhail, 2009). Additionally, dysregulation of peripheral glucose homeostasis increases AD risk and cognitive impairment (Wijesekara et al., 2018). Nevertheless, a prospective study showed little role of glucose intolerance in the pathogenesis of AD (Thambisetty et al., 2013). A systematic review and meta-analysis of preclinical and clinical studies showed that the expression of glucose transporters (GLUT1 and GLUT3) is decreased in the cerebral cortex and hippocampus. Nonetheless GLUT2 and GLUT12 are augmented in the cerebral cortex and hippocampus as a compensatory mechanism to reduce brain IR (Kyrtata et al., 2021).

Further, hypertension is also promoting AD neuropathology by increasing the deposition of Aβ peptides in brain tissues (Díaz-Ruiz et al., 2009). It has been demonstrated that mice chronically exposed to high blood pressure exhibit deposition of amyloid aggregates, the primary histological character of AD, and memory loss in particular tasks. Hypertensive challenge enhances the expression of the receptor for advanced glycated end products, resulting in Aβ accumulation and learning dysfunction (Carnevale et al., 2016). The development of hypertension is associated with AD risk (Ruthirakuhan et al., 2024). A systematic review and meta-analysis indicated that stage I systolic hypertension increases AD risk by 18%, while stage II systolic hypertension increases AD risk by 25%. Conversely, diastolic hypertension was not linked with AD risk (Lennon et al., 2019).

Thus, each component of metabolic syndrome contributes to AD neuropathology through multiple pathways suggesting the metabolic-cognitive syndrome which was proposed by Frisardi et al. (Frisardi et al., 2010). Collectively, these findings highlighted that metabolic syndrome is a risk factor for the induction and progression of AD by different cellular and molecular mechanisms that are not fully interpreted in clinical settings.

## Mechanistic Links Between Metabolic Syndrome and AD

### Brain IR and Glycogen Synthase Kinase 3 Beta (GSK3β)

The insulin receptor is present in many areas of both the developing and adult brain, and its functions have attracted the attention of current research (Plum et al., 2005; X. Zhang et al., 2022). Insulin enters the brain through the BBB via receptor-mediated transport to modulate food intake, sympathetic activity, and peripheral insulin action by the suppression of gluconeogenesis in the liver. Moreover, insulin suppresses neuronal apoptosis through activation of protein kinase B in vitro, as well as modulating phosphorylation of tau, metabolism of the APP, and clearance of Aβ from the brain in vivo (Plum et al., 2005). Moreover, brain insulin signaling is involved in the regulation of synaptic plasticity, dendritic outgrowth, learning, and memory (Chiu & Cline, 2010). Yanagita et al. found that dysregulation of brain insulin signaling is linked with the impairment in Aβ clearance and contributes to AD pathology (Yanagita et al., 2013). In addition, inactivation of insulin or insulin-like growth factor-1 receptors in the central amygdala and hippocampus results in cognitive impairment, anxiety-like behavior, and glucose intolerance in mice (Soto et al., 2019). Therefore, brain insulin/ insulin-like growth factor-1 signaling is important for higher neural processing and systemic metabolism.

Brain IR defined as the failure of brain cells to respond for insulin results in the impairments of synaptic functions (Hölscher, 2020; Spinelli et al., 2020). Brain IR is a hallmark of AD neuropathology and contributes to the impairment of hippocampal plasticity and the development of cognitive impairment (Bayram et al., 2022; Hölscher, 2020; Spinelli et al., 2020). Brain IR is often associated with T2D leading to neuroinflammation, oxidative stress, neuronal apoptosis, and the development of neurodegeneration (Maciejczyk et al., 2019). There are no specific biomarkers to detect the level of brain IR. However, plasma exosomal biomarkers of brain IR, such as higher pSer312-insulin receptor substrate 1(IRS-1) (ineffective insulin signaling) and lower p-panTyr-IRS-1 (effective insulin signaling), are linked with brain atrophy in AD and reflect regional IRS-1 expression. In addition, AβPP-Aβ CSF-based panels could provide more information about the brain IR status and progression of neurodegeneration (Mullins et al., 2017a, 2017b). Augmented levels of leptin and decreased levels of peptide tyrosine tyrosine are features of peripheral IR in obesity, and similar abnormalities in AD brains reveal that peptide tyrosine tyrosine and leptin levels could also be markers of brain IR (Chetram Deochand, 2013).

In addition, peripheral IR in T2D and metabolic disorders can induce brain IR through TNF-α signaling (De Felice & Ferreira, 2014). However, brain IR may develop independently in AD due to the progressive accumulation of Aβ and NFTs regardless of ApoE4 status or peripheral blood glucose (Talbot et al., 2012). Besides, the CNS has a significant impact on the regulation of peripheral insulin sensitivity and glucose homeostasis through regulation of liver, brown adipose tissue, and pancreatic function, which are regulatory pathways implicated in T2D and obesity (Ruud et al., 2017). Therefore, there is a close relationship between peripheral and brain IR.

**Fig. 3:**
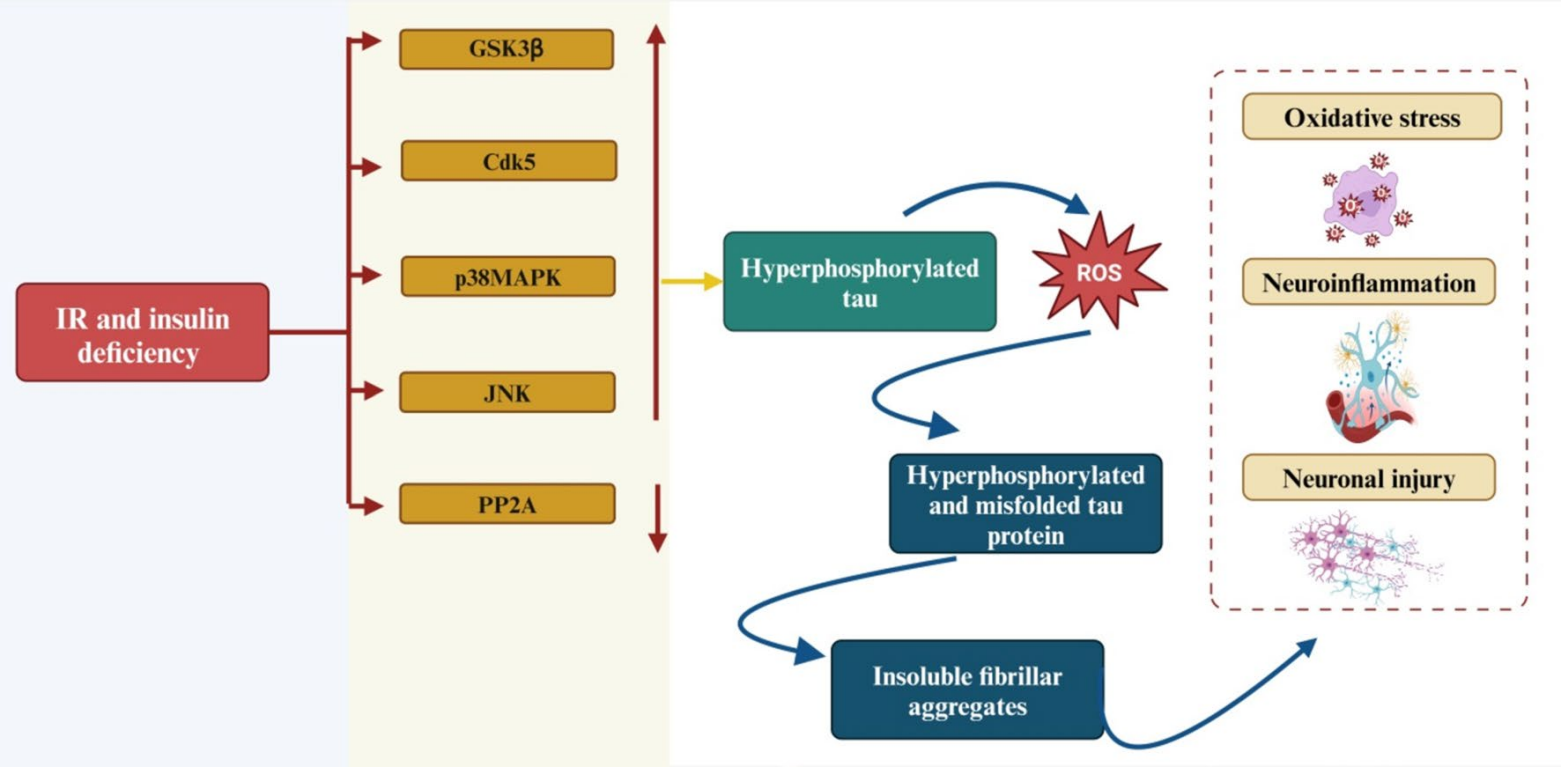
Brain IR and AD neuropathology: IR and insulin deficiency trigger the activation of GSK3β, Cdk5, p38MAPK, and JNK and inhibition of PP2A, leading to tau protein hyperphosphorylation, which induces the production of ROS. These changes lead to the aggregation of insoluble fibrils, which induce the development of oxidative stress, neuroinflammation, and neuronal injury. “Created in BioRender. Alexiou, A. (2025) https://BioRender.com/m22k703”

In addition, brain IR promotes the accumulation and inhibits the clearance of Aβ as well as the development of NFTs (Mullins et al., 2017a, 2017b). Thus, brain IR is developed in early AD and contributes to progressive neurodegeneration. Furthermore, brain IR results in abnormal neuronal glucose metabolism and neuronal energy, leading to cognitive impairment and memory loss (Daulatzai, 2017). According to these findings, AD was proposed as type 3 diabetes due to abnormal insulin signaling and glucose metabolism in the brain (Michailidis et al., 2022). Different studies disclosed that brain IR promotes AD neuropathology by inducing APP expression, hyperphosphorylation of tau protein, neuronal oxidative stress, mitochondrial dysfunction, ER stress, and the development of neuroinflammation (Pedersen & Flynn, 2004; Vandal et al., 2014). Likewise, brain IR suppresses specific genes involved in the regulation of synaptic plasticity and cholinergic neurotransmission, causing neurocognitive impairment (Pan et al., 2023). Moreover, brain IR triggers the activation of GSK3β, cyclin-dependent kinase 5 (Cdk5), p38 mitogen-activated protein kinase (p38MAPK), and Janus N-terminal kinase (JNK) and inhibition of protein phosphatase 2A (PP2A), resulting in hyperphosphorylation of tau protein (M. De La Monte 2012). Increased oxidative stress causes ROS production and ubiquitination, which then leads to tau misfolding. Misfolded tau aggregates create insoluble twisted fibrils that exhibit neurotoxicity and mediate neuroinflammation, oxidative stress, and neuronal injury (M. De La Monte 2012) (Fig. 3).

**Fig. 4:**
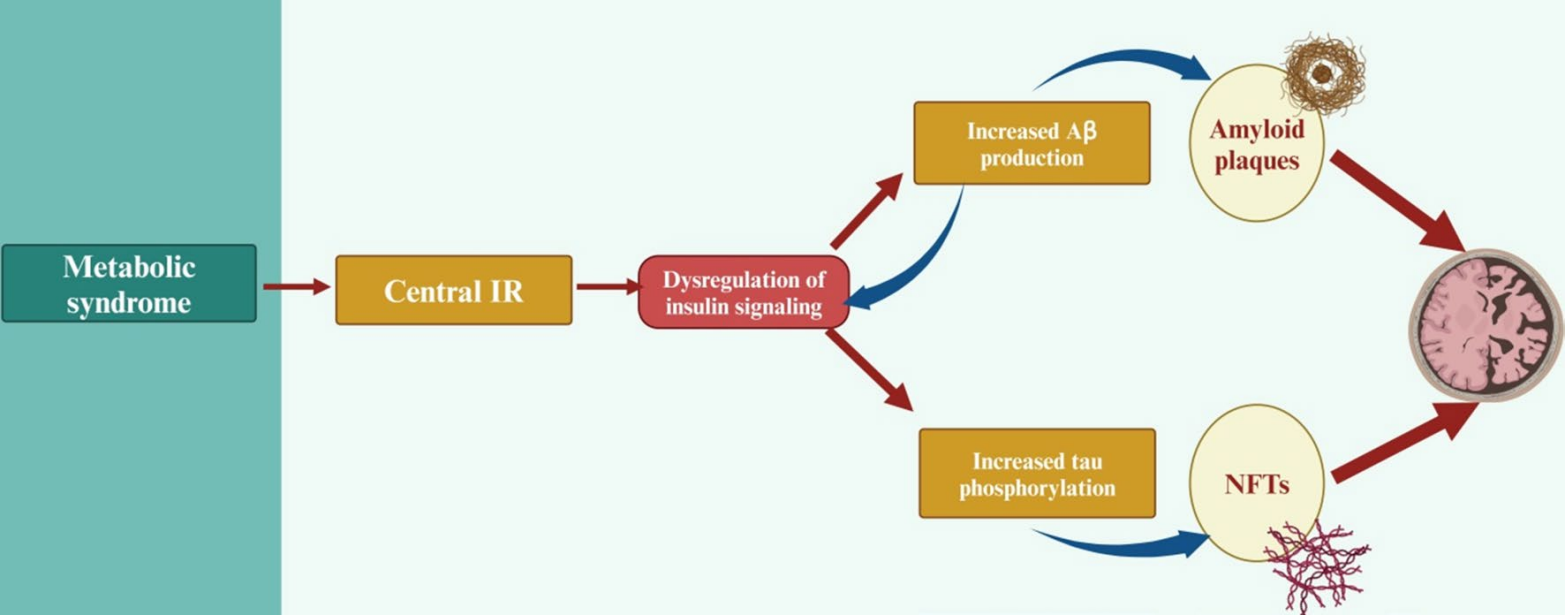
Metabolic syndrome and AD pathology in the presence of IR: Metabolic syndrome through induction of brain IR provokes dysregulation of brain insulin signaling, which induces the production of Aβ and hyperphosphorylation of tau protein with the formation of amyloid plaque and NFTs, respectively. “Created in BioRender. Alexiou, A. (2025) https://BioRender.com/j78k463”

On the other hand, metabolic syndrome through induction of chronic low-grade inflammatory status can induce peripheral IR and brain IR (Muzurović et al., 2021). A population study disclosed that IR may underlie cortical brain atrophy associated with metabolic syndrome (Lu et al., 2021). Moreover, microstructural white matter alterations are correlated with cognitive decline in adults with metabolic syndrome (Alfaro et al., 2016). Peripheral metabolic syndrome stimulates central IR in the brain. The resulting impaired insulin signaling, which primarily affects the phosphoinositide 3-kinase/Akt pathway, leads to elevated APP processing/Aβ levels and tau phosphorylation. Finally, elevated Aβ further impairs insulin signaling to trigger AD neuropathology as well as cognitive loss (Kim & Feldman, 2015). Therefore, metabolic syndrome can trigger AD neuropathology by increasing Aβ formation and hyperphosphorylation of tau protein (Fig. 4).

**Fig. 5:**
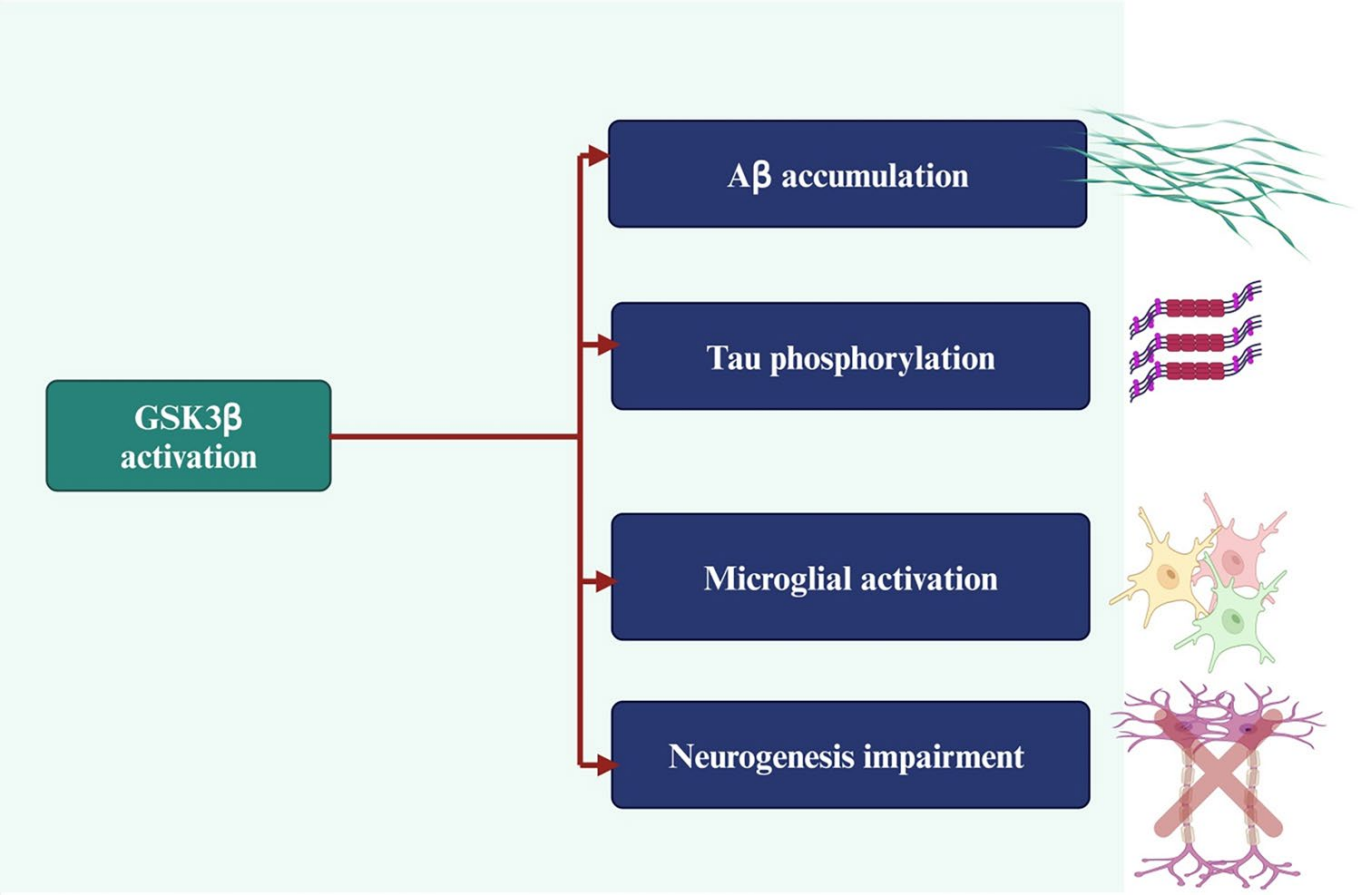
GSK3β overactivity and AD neuropathology: Overactivity and overexpression of GSK3β are associated with the development and progression of AD through the induction of the deposition of Aβ and tau protein, microglial activation, and inhibition of neurogenesis. “Created in BioRender. Alexiou, A. (2025) https://BioRender.com/x57c140”

GSK3 is a serine/threonine protein kinase that controls glycogen synthesis and involved in the regulation of blood glucose and cellular metabolism (Rayasam et al., 2009). Phosphorylation of Tyr216 and Ser9 regulates GSK3β activity positively and negatively, respectively. Whereas phosphorylation of the residue Tyr216 occurs at the GSK3β translation process and leads to the synthesis of the fully activated kinase, Ser9 phosphorylation tends to be the primary regulatory alteration during the enzyme’s lifespan. GSK3β remains inhibited when phosphorylated at Ser9, but when dephosphorylated, the kinase is activated (Duda et al., 2020). The GSK3β is the most common type of GSK3, highly expressed in the brain (Duda et al., 2020; Engel et al., 2018) and is intricate in the regulation of synaptic plasticity and neurogenesis through phosphoinositide 3-kinase/AKT and Wnt/β-catenin signaling pathways (Y. Wang et al., 2021; Zheng et al., 2017; Zwamborn et al., 2018). GSK3β is also involved in the neurodegeneration by down-regulating the antioxidant cell defense induced by nuclear factor erythroid 2-related factor 2 (Nrf2) (Rojo et al., 2008). It has been shown that abnormalities in insulin and insulin-like growth factor type I and II signaling mechanisms in brains with AD were correlated with increased GSK3β activity (Steen et al., 2005). Findings from a preclinical study showed that high-fat diet-induced cognitive impairment is mediated by stimulating neuronal GSK3β activity, which causes oxidative stress, mitochondrial dysfunction, and neuroinflammation (Wohua & Weiming, 2019). The expression of GSK3β is augmented in the brains of AD patients before the deposition of NFTs suggesting that GSK3β might be a primary event involved in AD neuropathology (Leroy et al., 2007). Moreover, genes encoding of GSK3β and comparable tau kinases could alter genetic risk for Aβ pathology. Therefore, combined variation in *GSK3β* and *APP*-related genes may lead to elevated amyloid burden (Hohman et al., 2014). In addition, GSK3β activity in the platelets is augmented and correlated with cognitive impairment and disease severity in AD patients (Pláteník et al., 2014). Besides, exaggeration of neuronal GSK3β activity is associated with Aβ accumulation and tau protein hyperphosphorylation through induction of APP processing and neuronal oxidative stress. In addition, GSK3β overactivity is associated with microglial activation and inhibition of neurogenesis, resulting in cognitive impairment (Lauretti et al., 2020) (Fig. 5).

Moreover, GSK3β overactivity may develop due to IR, T2D, and obesity (Chauhan et al., 2015; Ying Zhang et al., 2018). High-fat diet-induced IR and moderate visceral fat gain in rats are mediated by activating GSK3β (Henriksen et al., 2008). In addition, inhibition of brain GSK3β improves neuronal IRS-1, which reduces central sympathetic outflow and decreases blood pressure, proposing that aberrant GSK3β expression in the brain is implicated in the pathologic process of central hypertension (Cheng et al., 2015). These findings indicated that metabolic syndrome may induce AD neuropathology through induction the expression of brain GSK3β.

### Oxidative Stress and Mitochondrial Dysfunction

**Fig. 6:**
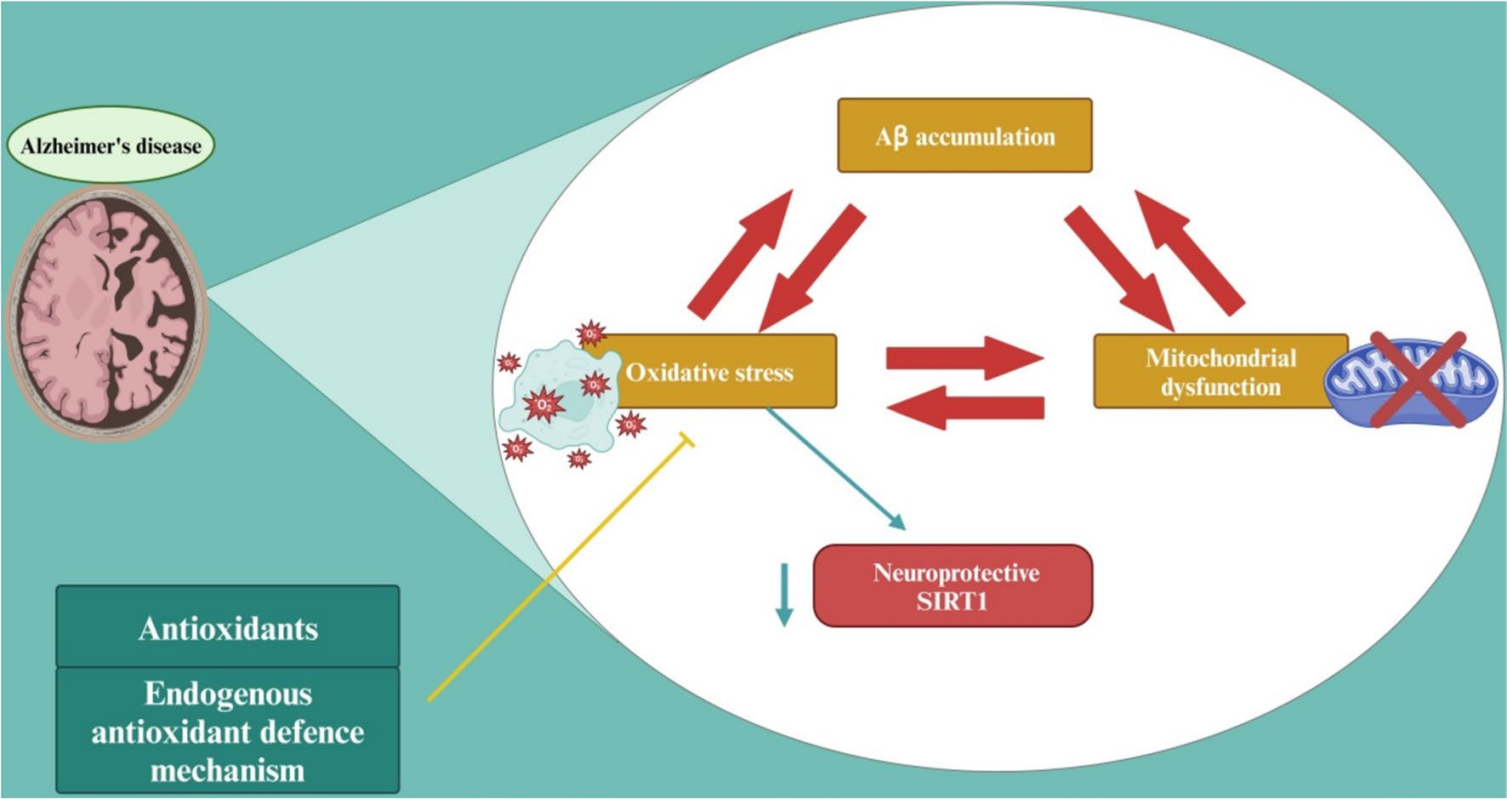
Oxidative stress and mitochondrial dysfunction in AD: Progressive accumulation of Aβ triggers the development of oxidative stress and mitochondrial dysfunction that cause AD. Oxidative stress can reduce the expression of the neuroprotective SIRT1. Targeting oxidative stress by antioxidants or modulation of endogenous antioxidant defense mechanisms can mitigate AD neuropathology by reducing oxidative stress and mitochondrial dysfunction. “Created in BioRender. Alexiou, A. (2025) https://BioRender.com/m94f166”

Oxidative stress and mitochondrial dysfunction are implicated in the pathogenesis of AD by inducing lipid peroxidation of the neuronal membrane and impairment of neuronal energy homeostasis (Misrani et al., 2021; X. Wang et al., 2014a, 2014b). Moreover, it was reported that mitochondrial dysfunction is triggered by oxidative stress and also amplify ROS formation, possibly resulting in the elevated oxidative stress in AD in a vicious downward spiral manner (Yan et al., 2013). It has been shown that the biomarkers of mitochondrial dysfunction and oxidative stress are augmented in the CSF and brain areas in AD (Bhatia & Sharma, 2021). Additionally, oxidative stress and mitochondrial dysfunction promote AD neuropathology by increasing APP processing and generation of neurotoxic Aβ, which also induce the neuronal mitochondrial dysfunction and oxidative stress (Park et al., 2020). Furthermore, oxidative stress may induce AD neuropathology indirectly by reducing the expression of the neuroprotective sirtuin (SIRT)1, which improves neurogenesis and reduces neuroinflammation in different neurodegenerative diseases (Batiha et al., 2023). It has been established that SIRT3 levels are remarkably reduced in AD, leading to elevations of ROS accumulation and neuronal damage. As well, SIRT3 reduction promotes p53-mediated mitochondrial dysfunction in AD. Therapeutic regulation of SIRT3 activity may ameliorate mitochondrial dysfunction and neurodegeneration in AD (J. Lee et al., 2018). Furthermore, antioxidant agents could be of therapeutic significance in the management of neurodegenerative diseases including AD (Fadaka et al., 2019). A recent study demonstrated that paeonol can ameliorate cognitive disturbances in a rat model of AD by reducing oxidative stress and mitochondrial dysfunction (Tayanloo-Beik et al., 2022). These findings are consistent with Manickam Rajkumaret al., who reported that reducing oxidative stress and mitochondrial dysfunction could reverse AD pathology in a rat model via treatment with chitosan-polylactic acid-loaded magnesium oxide nanocomposite (Rajkumar et al., 2023). Thus, modulation of endogenous antioxidant defense mechanism is an integral method to reduce oxidative stress and improve cell stress response in neurodegenerative diseases by increasing the expression of SIRT1 and Nrf2 (Calabrese et al., 2010). Recently, it was reported that S-nitrosoglutathione suppressed oxidative stress and improved the cognitive deficits and AD pathological conditions in a rat model of sporadic AD via activating the Nrf2 antioxidant signaling pathway (Dubey et al., 2024). In addition, brain nitric oxide can provide a neuroprotection by inducing the expression of haemoxygenase 1 (Calabrese et al., 2007). Consequently, stimulation of vitagenes against oxidative stress could be a novel therapeutic strategy against cancer and neurodegenerative diseases (Calabrese et al., 2009; Renis et al., 1996). Thus, activation of brain antioxidant enzymes may reduce oxidative stress and mitochondrial dysfunction that linked with AD neuropathology (Fig. 6).

Furthermore, oxidative stress and mitochondrial dysfunction are often associated with the pathophysiology of metabolic syndrome (Bhatti et al., 2017). These cellular changes in the metabolic syndrome can induce the development of brain IR and the progression of AD (De La Monte & Tong, 2014; Galizzi & Di Carlo, 2022). Peripheral IR in the metabolic syndrome is correlated with increased ceramide production due to the elevated supply of fatty acids derived from a high-fat diet (Haus et al., 2009; Summers, 2006). Remarkably, ceramide like other neurotoxic lipids passes through the BBB and triggers brain IR (Lyn-Cook et al., 2009; Tong & De La Monte, 2009). Additionally, cytotoxic ceramides in the brain trigger oxidative stress and mitochondrial dysfunction, resulting in neuronal death (Galizzi & Di Carlo, 2022; M. De La Monte 2012). Notably, SIRT1 signaling is highly reduced in the metabolic syndrome, and activation of SIRT1 can protect against metabolic syndrome-induced neurodegeneration (Caron et al., 2014). A previous experimental study demonstrated that ablation of the Nrf2 gene in adipocytes results in the development of severe metabolic syndrome in mice. The study revealed that Nrf2 in adipocytes plays a significant role in improving insulin resistance via upregulating antioxidant gene expression, resulting in a reduction in cellular ROS (Branca et al., 2017). Moreover, a previous study demonstrated that high-fat diet-induced obesity accompanied with IR involves Nrf2 suppression (Abo El-Magd et al., 2018). Nrf2 has a potent neuroprotective role against the development of neurodegenerative diseases by counteracting oxidative stress and mitochondrial dysfunction and improving cognitive function in AD (Dinkova‐Kostova et al. 2018; Tian et al., 2019). Genetic deletion of the *Nrf2* gene in transgenic mice overexpressing APP triggers severe cognitive dysfunction and impairment of spatial learning ability due to progressive accumulation of Aβ and associated synaptic failure (Branca et al., 2017). Preclinical and clinical findings showed that phosphorylated Nrf2 level is increased in human peripheral blood cells of AD patients and in an AD mouse model at various stages as a compensatory mechanism to reduce oxidative stress in AD (Mota et al., 2015). Hence, oxidative stress and mitochondrial dysfunction in the metabolic syndrome adversely affect AD neuropathology.

### Neuroinflammation and Inflammatory Signaling Pathways

Neuroinflammation is a specific immune response of the CNS to stress or exogenous stimuli. Acute neuroinflammation has a neuroprotective effect by eliminating the causative factors, though chronic neuroinflammation provokes progressive neurodegeneration by inducing derangement of the BBB and inhibiting of neuronal homeostasis (Hopper et al., 2012; Lyman et al., 2014; Takata et al., 2021). It has been observed that neuroinflammation exacerbates AD neuropathology by different mechanisms, such as induction of synaptic/neuronal loss, inhibition of neurogenesis, and triggering of neuronal apoptosis (Bassani et al., 2017; Lecca et al., 2022; L. Zhang et al., 2021). In transgenic mice overexpressing APP/PS1, neuroinflammation and exaggerated inflammatory signaling pathways result in acute cognitive impairment through dysregulation of hippocampal synaptic plasticity (Lopez‐Rodriguez et al. 2021). A cohort study illustrated that neuroinflammation and cerebrovascular dysfunction are early events developing at pre-symptomatic stages of AD and are linked to its progression (Janelidze et al., 2018). These findings highlighted that neuroinflammation is assumed to be a potential mechanism intricately involved in the pathogenesis of AD. Furthermore, metabolic syndrome through low-grade inflammatory disorders and activated inflammatory signaling may induce neuroinflammation (Purkayastha & Cai, 2013; Więckowska-Gacek et al., 2021). In addition, IR and leptin resistance are linked to the development of neuroinflammation (Komleva et al., 2021; Mejido et al., 2020). Thus, metabolic syndrome-induced neuroinflammation could be the most important mechanistic pathway in the development and progression of AD.

**Fig. 7:**
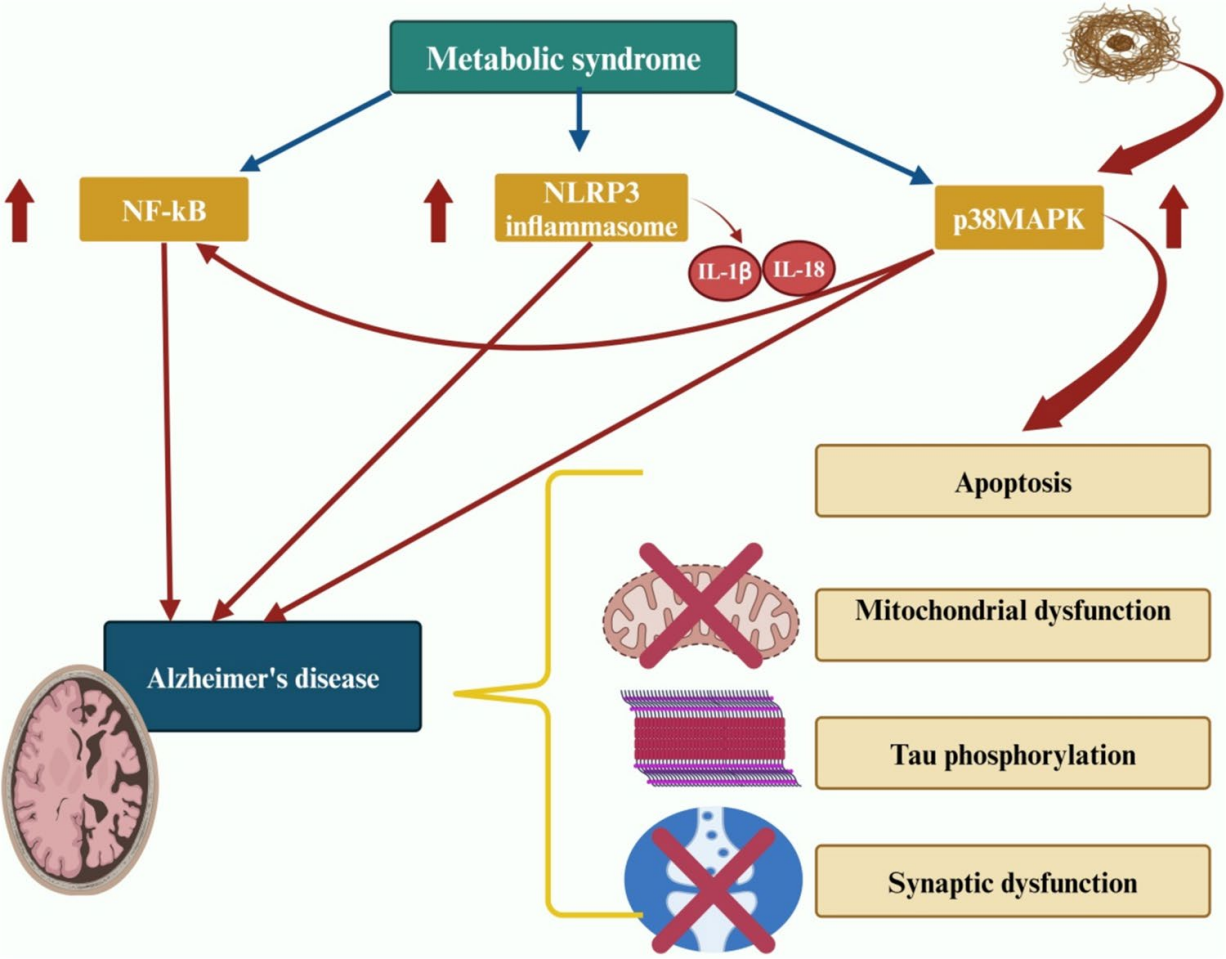
Inflammatory signaling pathways probably linking metabolic syndrome and AD: Inflammatory signaling pathways, including p38MAPK, NLRP3 inflammasome, and NF-κB, are implicated in the pathogenesis of AD. The activation of p38MAPK by Aβ plaques can lead to mitochondrial dysfunction, apoptosis, tau phosphorylation, and synaptic dysfunction. p38MAPK exhibits the ability to activate NF-κB. The activation of NLRP3 produces pro-inflammatory cytokines IL-1β and IL-18. These inflammatory signaling pathways are also exaggerated in the metabolic syndrome, which could be a potential link between metabolic syndrome and AD. “Created in BioRender. Alexiou, A. (2025) https://BioRender.com/f73m105”

Of note, inflammatory signaling such as p38MAPK, nod-like receptor pyrin 3 (NLRP3) inflammasome, and nuclear factor kappa B (NF-κB) are intricate in the pathogenesis of neurodegenerative disorders (Alrouji et al., 2023; Jha et al., 2023; Kheiri et al., 2018). The NF-κB signaling is increased in AD patients compared to healthy controls (Huang et al., 2005). Exaggeration of NF-κB in AD is due to the accumulation of Aβ through oxidative stress-dependent mechanism (Azargoonjahromi, 2024; Rather et al., 2021). In addition, inhibition of NLRP3, considering its pivotal role in Aβ- and tau-mediated pathological events, is undoubtedly a promising approach for developing treatments for AD (Van Zeller et al., 2021). The activation of NLRP3 promotes the occurrence of AD through producing IL-1β, IL-18, and other cytokines (Bai & Zhang, 2021). Likewise, p38MAPK is also implicated in neuroinflammation and AD due to its ability to activate NF-κB (Kheiri et al., 2018). Aβ plaques induce neuronal damages such as mitochondrial dysfunction, apoptosis, tau phosphorylation, and synaptic dysfunction through the activation of p38 MAPK (J. K. Lee & Kim, 2017). Furthermore, the inflammatory signaling pathways are also exaggerated in the metabolic syndrome. For example, nutrient excess and low-grade inflammatory reactions in the metabolic syndrome provoke the activation of NF-κB, p38MAPK, and NLRP3 inflammasome signaling (Asrih et al., 2013; Catrysse & Van Loo, 2017; F. Wang & Mo, 2020). Consequently, inflammatory signaling pathways which are upregulated in the metabolic syndrome may induce AD neuropathology through the propagation of systemic inflammation. Therefore, inflammatory signaling pathways could be a potential link between metabolic syndrome and AD (Fig. 7).

### Proteostasis Homeostasis

The homeostasis of cellular proteins, or proteostasis, is essential for neuronal function and brain processes, such as learning and memory (Cozachenco et al., 2023a, 2023b). Proteostasis includes the regulatory processes that preserve the proteome balance via functions including protein synthesis, folding, degradation, and catabolism, which are regulated by pathways like the ubiquitin–proteasome system and autophagy (Lin & Ho, 2024). There is growing evidence that impaired proteostasis is linked to the advancement of neurodegenerative illnesses, particularly AD (Thapa et al., 2024). Proteostasis consists of a set of cellular mechanisms that regulate protein synthesis, folding, post-translational modification, and degradation, all of which are disrupted in AD. Notably, dysregulation of proteostasis contributes significantly to synapse dysfunction and memory impairment, which are the primary clinical manifestations of AD (Cozachenco et al., 2023a, 2023b). Autophagy acts as a proteostasis process by removing protein clumps, but it gradually diminishes with age and AD, allowing harmful proteins to accumulate and triggering neurodegeneration. Under normal conditions, autophagy eliminates aberrant proteins and damaged organelles, while any disruption in this process can exacerbate amyloid and tau pathology, especially in AD. There is increasing focus on therapeutic strategies that promote autophagy, involving decreased calorie intake, autophagy-stimulating medications, and genetic therapy (Barmaki et al., 2023). Recent evidence indicates that disrupting non-canonical autophagy in microglia can impair the capacity to remove Aβ, resulting in progressive neurodegeneration in a mouse model of AD (Z. Wang et al., 2023).

On the other hand, defective proteostasis is also implicated in the pathogenesis of metabolic syndrome. It has been shown that chronic oxidative state, lipid peroxidation, protein oxidation, production of advanced glycation end products, glycosylation, endoplasmic reticulum stress, and liberation of cytokines in metabolic syndrome influence the highly redox-regulated ubiquitin-proteasomal system (Höhn et al., 2016). Increasing evidence suggests that defective autophagy resulting from metabolic syndrome is associated with oxidative stress, inflammation, and foam cell production, hence exacerbating atherosclerosis and other cardiometabolic disorders (J. Xu et al., 2021). The role of autophagy in metabolic disorders has been widely studied using genetic models that showed diverse metabolic features. For example, mice with a knockout of *Atg7*, an essential autophagy gene in pancreatic β-cells producing insulin, showed structural and functional defects of pancreatic β-cells, resulting in glucose intolerance and susceptibility to diabetes in the presence of metabolic stress (Quan et al., 2012). Conversely, autophagy knockout in skeletal muscle cells resulted in the stimulation of fibroblast growth factor 21 as a mitokine in response to mitochondrial stress, as well as resistance to diet-evoked obesity and IR. In contrast to the expectation that autophagy deficiency associated with mitochondrial dysfunction in insulin target tissues would lead to IR (H. Lim et al., 2018). Systemic autophagy insufficiency of physiologically relevant degree rather than tissue-specific knockout compromised adaptation to metabolic stress and facilitated progression from obesity to T2D (Y.-M. Lim et al., 2014). Furthermore, overexpression of Atg5, another essential autophagy gene, improved the metabolic profile of aged mice (Pyo et al., 2013). These findings indicate that systemically increased autophagic activity may exhibit a positive impact on body metabolism during metabolic stress (H. Lim et al., 2018). Therefore, defective proteostasis could be a central mechanism linking AD and metabolic syndrome.

### Lipid Mediators

Bioactive lipids modulate significant functions of neural membrane biology, such as protein–lipid interactions, trans-membrane, and trans-synaptic signaling. But, a number of highly reactive prostaglandins (PGs), lipoxin A4 (LXA4), free fatty acids, lysophospholipids, eicosanoids, platelet-activating factor, and ROS, all of which are produced by increased phospholipase A2 (PLA2) activity and arachidonic acid release, contribute to cellular injury, notably in neurodegeneration. PLA2 stimulation and PG synthesis are among the first initiating processes in promoting brain-damage pathways, which may result in long-term neurologic impairments. Altered membrane-associated PLA2 activity has been correlated to a wide range of acute and chronic brain injuries, including cerebral trauma, ischemic damage, induced seizures and epilepsy, schizophrenia, and, particularly, AD. Animal models and brain cells in culture stimulated with PLA2 inducers, PGs, cytokines, and correlated lipid mediators have been extensively investigated to explore the biochemical mechanisms of PLA2 overactivation and its pathological effects on CNS structure and function. Furthermore, the expression of both cyclooxygenase-2 and PLA2 shows to be considerably activated during AD, demonstrating the significance of inflammatory gene pathways as a consequence of brain injury (Bazan et al., 2002). Furthermore, LXA4, a lipid mediator of inflammation resolution reported to enhance endocannabinoid signaling in the brain, is decreased in the aging brain. Genetic inhibition of 5-lipoxygenase, the enzyme mediating LXA4 synthesis, triggers learning impairment in mice. In contrast, administration of exogenous LXA4 suppressed cytokine formation and memory loss caused by inflammation in mice. In addition, the CSF LXA4 level is decreased in patients with dementia and significantly correlated with cognitive function, brain-derived neurotrophic factor, and AD-linked Aβ. Thus, decreased LXA4 levels may contribute to responsiveness to age-related cognitive disorders, and triggering LXA4 signaling may constitute a promising approach to avoid early cognitive impairment in AD (Pamplona et al., 2022). Moreover, dysregulation of PGs is intricate in the development and progression of metabolic syndrome. Findings from a preclinical study confirm that low-grade chronic inflammation and exaggerated prostanoid pathway are correlated with obesity-related dyslipidemia, abdominal obesity, and IR (Pawelzik et al., 2019). However, negative correlations were identified between metabolic syndrome and LXA4 levels in periodontal disease patients. This finding demonstrates the protective impact of the proresolving lipid mediator LXA4 in the connection between periodontal disorder and metabolic syndrome (Doğan et al., 2019). Thus, dysregulation of lipid mediators in metabolic syndrome contributes to the development of AD.

Collectively, current evidence from epidemiological, neuroimaging, pathological, pharmacotherapeutic, and clinical studies indicate an association of AD with metabolic syndrome either in isolation or in aggregate. In the light of this evidence, clinician may consider lifestyle interventions toward an early and effective cardiovascular risk-factor management to reduce the cardiometabolic and the cognitive decline risk, while further research of other preventive strategies may be warranted.

Interestingly, strengthening AD conceptualization, modeling, and evaluation is critical for developing effective therapies. Addressing unanswered concerns with the suggested conceptual may accelerate the development of useful disease-modifying approaches for AD (Cozachenco et al., 2023a, 2023b). In addition, studies have established that physical activity can enhance general brain health, potentially delaying or alleviating AD-correlated cognitive impairments and pathology. Physical activity affects cognitive function, vascular health, and brain metabolism; all of these assist the aging population, involving AD patients (Maliszewska-Cyna et al., 2016). Therefore, new perspectives into the effect of non-pharmacologic strategies in the modulation of AD neuropathology, which may provide the potential of enhancing quality of life by diminishing cognitive decline and incident AD in patients with metabolic syndrome, are needed.

## Conclusions

Metabolic syndrome through the induction of brain IR can trigger AD neuropathology by increasing Aβ formation and hyperphosphorylation of tau protein, leading to the generation of amyloid plaques and NFTs, respectively. Oxidative stress and mitochondrial dysfunction together with dysregulation of different signaling pathways such as SIRT1 and Nrf2 adversely affect AD neuropathology. Furthermore, inflammatory signaling pathways that are upregulated in the metabolic syndrome may induce AD neuropathology through the propagation of systemic inflammation. In addition, metabolic syndrome-induced neuroinflammation could be the most important mechanistic pathway in the development and progression of AD. Finally, defective proteostasis as well as dysregulation of lipid mediators could be probable mechanisms linking AD and metabolic syndrome.

Overall, metabolic syndrome is perceived as a possible risk factor for the induction of AD neuropathology by diverse signaling pathways such as the induction of brain IR, activation of inflammatory signaling cascades, neuroinflammation, defective proteostasis, and dysregulation of lipid mediators the link between metabolic syndrome and AD still unresolved, therefore, large-scale prospective and longitudinal studies are recommended in this aspect.

## Data Availability

No datasets were generated or analyzed during the current study.

## Abbreviations

AD: Alzheimer’s disease
ADDLs: Amyloid β-derived diffusible ligands
ApoE: Apolipoprotein E
APP: Amyloid precursor protein
Aβ: Amyloid beta
Cdk5: Cyclin-dependent kinase 5
CSF: Cerebrospinal fluid
GLUT: Glucose transporter
GSK3β: Glycogen synthase kinase 3 beta
HDL: High-density lipoprotein
IL: Interleukin
IR: Insulin resistance
IRS-1: Insulin receptor substrate 1
LXA4: Lipoxin A4
NFTs: Neurofibrillary tangles
NF-κB: Nuclear factor kappa B
NLRP3: Nod-like receptor pyrin 3
Nrf2: Nuclear factor erythroid 2-related factor 2
p38MAPK: P38 mitogen-activated protein kinase
PGs: Prostaglandins
PLA2: Phospholipase A2
PP2A: Protein phosphatase 2A
RAS: Renin-angiotensin system
ROS: Reactive oxygen species
sAPPα: Soluble amyloid precursor protein alpha
Sirtuin: Sirtuin
T2D: Type 2 diabetes
TNF-α: Tumor necrosis factor alpha
